# Bite Force in Elderly with Full Natural Dentition and Different Rehabilitation Prosthesis

**DOI:** 10.3390/ijerph18041424

**Published:** 2021-02-03

**Authors:** Licia Manzon, Iole Vozza, Ottavia Poli

**Affiliations:** 1 Department of Cardiovascular, Respiratory, Nephrologic, Anesthesiologic and Geriatric Sciences, Sapienza University of Rome, 00161 Rome, Italy; licia.manzon@uniroma1.it (L.M.); ottavia.poli@uniroma1.it (O.P.); 2 Department of Oral and Maxillofacial Sciences, Sapienza University of Rome, 00161 Rome, Italy

**Keywords:** natural dentition, oral rehabilitation, prosthesis, elderly

## Abstract

(1) Background: This study aimed to investigate maximum bite force (MBF) in elderly patients with natural full dentition (FD), patients rehabilitated with Traditional Complete Dentures (CD), with overdentures (IRO) and edentulous patients (ED). We also tested whether MBF changes are associated with gender, age of the patients and body mass index (BMI) as result of altered food; (2) Methods: Three hundred and sixty-eight geriatric patients were included. We studied two types of prostheses: (a) IRO with telescopic attachments. (b) CD (heat polymerized polymethyl methacrylate resin). The MBF was measured using a digital dynamometer with a bite fork; (3) Results: We found that MBF is higher in males than females, regardless of teeth presence or absence (*p* < 0.01). In patients with CD or IRO, there are no differences between males and females; prostheses improve MBF compared to edentulous patients (*p* < 0.0001) and this effect is greater with IRO prostheses (*p* < 0.0001); the chewing force of FD subjects remains greater (*p* < 0.0001); there are no differences among chewing strength based on different BMI categories, although FD subjects have a reduced incidence of obesity; there is a significant negative correlation between MBF and age (*p* = 0.038; R = 0.145), and no correlation between MBF and BMI; (4) Conclusions: This study showed that MBF improves more in patients using IRO prostheses, although not reaching the MBF of FD subjects. MBF does not correlate with BMI, although we found increased percentages of obesity in edentulous subjects or those with prostheses. Thus, old people wearing prostheses require special attention by a nutritionist to avoid risk of malnutrition.

## 1. Introduction

The elderly represents 20% of the population and this percentage tends to grow more and more [1]. Among the problems related to the elderly, a reduced masticatory force is the cause of several metabolic disorders. Masticatory or bite force is decreased in the elderly for a number of reasons. Some authors correlated the deterioration of the occlusal conditions and the lack of teeth in elderly subjects with the number of falls [2] and their psychiatric state [3].

Reduced oral hygiene, due to decreased manual dexterity, further promotes decays, periodontal diseases [4] and tooth loss until edentulism.

A consequence of decreased bite force is that chewing requires more masticatory cycles, the food is not adequately mushed, and coarser food particles are swallowed. In such conditions, people may opt for soft food, carbohydrates, fats and sweet foods, excluding proteins, fibers and vegetables. This type of diet results in malnutrition and increased risk of frailty development. Some studies [5,6] showed a relation between teeth loss and body mass index (BMI), a measure of body fat based on height and weight. In addition, such food regimes may favor the insurgence of diabetes and other metabolic diseases. People with full dentition (FD) maintain masticatory efficiency and a varied diet [7,8], whereas edentulous subjects are not able to comminute particles, making swallowing and digestion difficult [9]. Therefore, maintenance of chewing efficiency in elderly people is crucial to reduce public health expenditure and enhance their quality of life.

Chewing efficiency depends on many factors, such as occlusal contact number, bite force, and masticatory muscle work to grind and cut the food [10]. The harder the food is, the more muscle activity is required [10]. The maximum bite force (MBF) is the most reliable index of occlusal force and it is used to assess the functional state of the masticatory system [7,11,12]. 

Force levels tend to decrease physiologically with age, due to muscular atrophy, as demonstrated by computed tomography [13]. However, other authors suggest that computed tomography changes in dentate elderly subjects are not particularly important and that the reduction in bite force is more related to the problem of missing teeth [14]. The worst situation obviously occurs in subjects who have lost all their teeth. In any case, these subjects need to be rehabilitated to reestablish masticatory performance and esthetic appearance and to avoid the risks of reduced food intake, malnutrition, and functional and psychological disorders.

Conventional Complete denture (CD) is the cheapest and most widespread rehabilitating prosthesis in edentulous patients, although patients wearing CD may suffer from insufficient retention and stability during masticatory function. The lack of stability may cause pain during chewing, due to denture compression and mobility, limiting an efficient muscle action [7,15]. The masticatory function is 2.5 times worse than that of dentate subjects [16], especially for wearers of traditional CD in the lower arch. Possible causes are the reabsorption and atrophy of alveolar ridge of edentulous alveolus due to teeth loss. Moreover, CD wearers claim a reduced taste and texture sensation due to the complete covering of the palate [5].

Another possibility to restore of the edentulous arch might be an implant-retained overdenture (IRO). One or two implants are enough to sustain the denture that is retained by telescopic attachments, locator, bar or ball. This option enhances oral function and quality of life as compared to conventional dentures [7,15]. Furthermore, as Elsyad et al. sustained, chewing efficiency of overdentures supported by four implants (with bar attachment) increased when compared to overdentures supported by two implants [15]. Moreover, several clinical studies have shown decreased chewing time, decreased particle size of the bolus ready for swallowing and enhanced MBF in subjects rehabilitated with dental implants [7,17].

At present, a limited number of studies has investigated bite force in elderly subjects in relation to factors such as the dentition, the type of prostheses, the BMI, the gender and the age of the patients. Thus, the goal of our study is to investigate and compare MBF and BMI in elderly (males and females) with natural full dentition (FD), patients rehabilitated with Traditional Complete Dentures (CD), with overdentures (IRO) and edentulous patients (ED).

Our hypothesis is that subjects with full dentition and/or IRO wearers may have greater MBF as compared to CD wearers and ED patients. In addition, we tested the hypothesis that MBF changes may be associated with the gender and age of the patients. Lastly, we investigated whether MBF changes are associated with changes of BMI (as a result of altered food regime) and whether people with greater MBF have a better nutritional status, according to BMI classification [18]. In fact, subjects with reduced MBF may restrict their diet to more soft food rich in carbohydrates sugar and fats, with risk of malnutrition and obesity.

## 2. Materials and Methods

### 2.1. Subjects

Three hundred and sixty-eight geriatric patients, 186 males and 182 females, were included in this study. Only patients over the age of 65 were included in the study, according to the common definition of “geriatric patient” [19]. The methodology and the goal of the study were explained and written informed consent signed by the patients was obtained before the beginning of the study. The study was approved by the Local Ethics Committee (1403/19).

### 2.2. Inclusion Criteria

- Subjects without problems, in everyday life, with natural dentition.
- Subjects wearing telescopic overdentures with 4 implants (2 in canine zone and two in premolar zone).
- Subjects wearing complete dentures in one arch (and natural teeth in the opposite arch) or both arches.
- All people were wearing their prosthesis at least for 1 year.
- No dental mobility or alveolar bone retraction ≤4.5 mm as measured by orthopantomography (OPT) [20].
- No implant mobility or pain or alveolar bone retraction around implants <1.5 mm measured by intraoral periapical radiographs.
- Absence of psychiatric diseases or movement problems.
- Lack of articular/muscular, dental or spontaneous pain.

### 2.3. Exclusion Criteria

- Poor oral hygiene.
- Periodontitis.
- Dental mobility.
- Teeth alveolar bone retraction >4.5 mm on OPT.
- Diabetes.
- Ongoing chemotherapy.

### 2.4. Prostheses

We studied wo types of prostheses:

(a) Implant-retained overdentures (IRO) with custom made telescopic attachments. The prosthesis substructure has been realized by TRINIA™ (Bicon. LLC, Boston, MA, USA) made up of interlaced multidirectional, multilayered fiberglass, immersed in a matrix of epoxy resin. Teeth have been realized in nanofiller composite by ^©^CANDULOR AG [21]. Telescopic attachments were preferred because they minimize movements of the distal portion of the denture and improve horizontal stability [22,23].

(b) Traditional complete dentures (CD) (heat polymerized polymethyl methacrylate resin, SINTODENT S.r.l., Rome, Italy). All resin denture teeth were obtained from Ivoclar Vivadent AG (Schaan, Liechtenstein).

All prostheses were made in the same laboratory, following the manufacturer’s guidelines.

### 2.5. Experimental Groups

Patients were divided into 5 groups:

1. Subjects with natural full dentition (FD) (*n* = 204).
2. Subjects wearing four implant-retained overdentures (two in canine zone and two in premolar zone) (IRO) (*n* = 40).
3. Subjects with natural teeth in one arch and wearing traditional complete dentures (CD) in opposite dental arch (CD/T) (*n* = 44).
4. Subjects wearing CD in both arches CD/CD (*n* = 40).
5. Edentulous subjects in both arches (ED) (*n* = 40; the same patients of group 4 after removal of the CD).

### 2.6. Body Mass Index

Height and weight were measured to the nearest 1 cm and 0.1 kg, respectively. BMI (body mass index) was calculated using the following formula: BMI = weight (kg)/height (m)^2^.

According to the WHO BMI cut-off diagnostic criteria, 18 patients were categorized as follows: <18.5 kg/m^2^, underweight; 18.5–24.9 kg/m^2^, normal-weight; 25.0–29.9 kg/m^2^, overweight; and ≥30 kg/m^2^, obese.

### 2.7. Measurement of Patients Maximum Bite Force (MBF)

The MBF was measured using a digital dynamometer (KRATOS Equipment model IDDKv4, seral number o7175142) with a bite fork. The bite fork was, each time, covered with a new latex finger to avoid contamination and, after its placement on the first molar zona, the patients were requested to bite as powerfully as possible on the device for 5 s, in upright position. The measure, reported in kg, has been repeated three times with two minutes interval in between and it was detected in both right and left arches. The instrumentation, methods, and operator were the same for all groups. The bite force was measured in the first molar area of all subjects where it is exerted 80% of the total bite force [24]. The mean of the left and right MBF values was calculated in each subject and used for the statistical analysis. The evaluation was performed by two prosthodontists who were blinded to the treatment. The MBF value for each subject was recorded and used for statistical analysis.

Patients’ MBF and BMI were measured after wearing the prosthesis for at least 1 year, since >5 months are necessary for functional adaptation to the new dentures, as suggested by Goiato and coworkers [25].

The instrumentation, methods, and operators were the same for all groups.

### 2.8. Statistical Analysis

Data were analyzed by ANOVA, considering gender, BMI and MBF as variables. Post hoc comparisons were performed using Fisher’s Protected Least Significant Difference (PLSD) test. 

Correlation analysis of MBF values with age and BMI was performed with simple regression analysis. Frequency distribution of weight categories among groups was assessed by chi-square test. 

The level of statistical significance was set at *p* < 0.05. Statistical analysis was performed using the Statview Software v.5.0.1 from SAS Institute (Bioz, Inc., Los Altos, CA, USA).

## 3. Results

### 3.1. Bite Force and Gender

**Figure 1:**
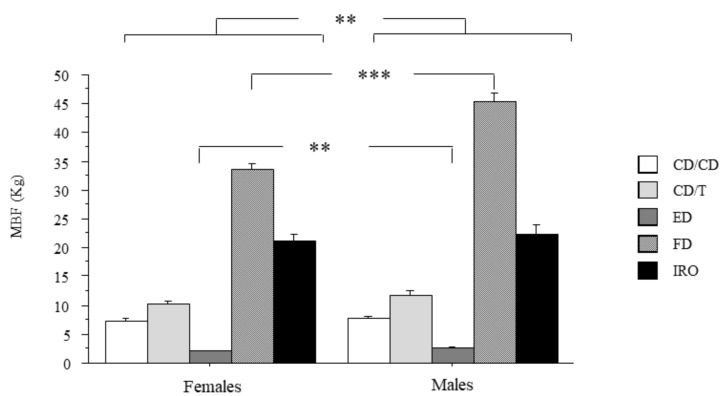
Bite force and gender. Maximal bite force according to gender. MBF: maximal bite force; subjects with natural full dentition (FD); subjects wearing implant-retained overdentures (IRO); subjects with natural teeth in one arch and wearing traditional complete dentures (CD) in opposite dental arch (CD/T); subjects wearing CD in both arches (CD/CD); edentulous patients in both arches (ED). Data are the mean ± SEM. Asterisk (*) indicates significant difference between the groups. ** *p* < 0.01; *** *p* < 0.001.

The bite force in males and females in the experimental groups is shown in Figure 1.

ANOVA showed a significant effect of gender (*p* < 0.01). Post hoc comparison showed that males had significantly higher bite force as compared to females (*p* < 0.0001). There was also a significant interaction of gender with the experimental groups (*p* = 0.001). Post hocs showed that males had greater MBF as compared to females in ED (*p* < 0.01) and FD (*p* < 0.0001) groups. In the groups of subjects wearing the prostheses, no differences in MBF between males and females were evidenced.

### 3.2. Bite Force and BMI

**Figure 2:**
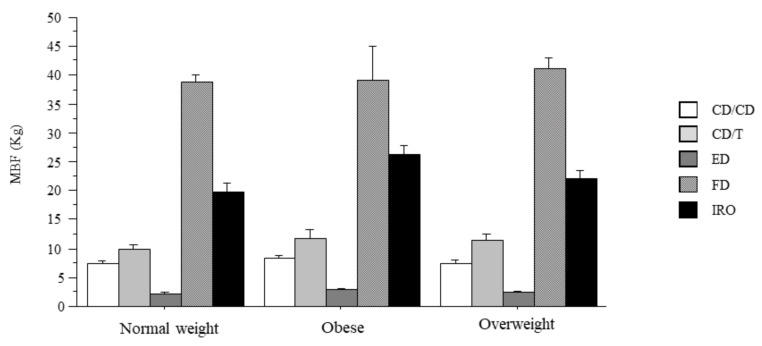
Bite force and body mass index (BMI). Maximal bite force according to body mass index classification. MBF: maximal bite force; subjects with natural full dentition (FD); subjects wearing implant-retained overdentures (IRO); subjects with natural teeth in one arch and wearing traditional complete dentures (CD) in opposite dental arch (CD/T); subjects wearing CD in both arches (CD/CD); edentulous patients in both arches (ED). Data are the mean ± SEM.

The bite force according to the BMI categories in groups is shown in Figure 2. Statistical analysis showed no significant effect of BMI on MBF (*p* = 0.494) and no interaction between BMI and experimental groups (*p* = 0.997). Nonetheless, the frequency distribution of weight categories among groups revealed that FD subjects have less incidence of obesity (5%) as compared to the other groups (ranging from 18 to 22%) (chi square-value: 24.48; *p* < 0.05).

### 3.3. Bite Force and Prothesis

**Figure 3:**
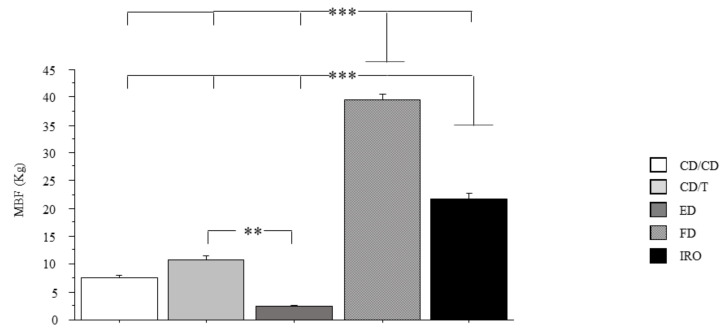
Bite force and prostheses. Maximal bite force in the three types of prostheses. MBF: maximal bite force; subjects with natural full dentition (FD); subjects wearing implant-retained overdentures (IRO); subjects with natural teeth in one arch and wearing traditional complete dentures (CD) in opposite dental arch (CD/T); subjects wearing CD in both arches (CD/CD); edentulous patients in both arches (ED). Data are the mean ± SEM. Asterisk (*) indicates significant difference between the groups. ** *p* < 0.01; *** *p* < 0.001.

The bite force according to the type of protheses is shown in Figure 3. There was a significant effect of the protheses on masticatory force (*p* < 0.0001). Subjects with full dentition (FD) had increased MBF as compared to CD/CD (*p* < 0.0001), CD/T (*p* < 0.0001), IRO (*p* < 0.0001), and ED (*p* < 0.0001). Subjects wearing IRO prostheses had increased MBF as compared to CD/CD (*p* < 0.0001), CD/T (*p* < 0.0001), and ED (*p* < 0.0001). Subjects with CD/T had increased MBF as compared to ED (*p* < 0.01). No differences in MBF between CD/CD and CD/T were found.

### 3.4. Correlation of Bite Force with Age

**Figure 4:**
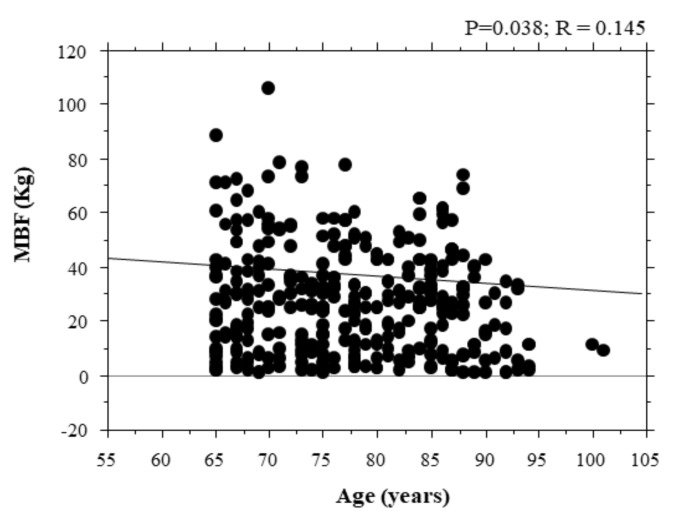
Correction of bite force with age. Correlations of maximal bite force with age of the subjects. MBF: maximal bite force; R = coefficient of correlation; P = *p*-value (*p*).

The correlation of bite force with age is shown in Figure 4. There was a negative correlation between bite force and age (*p* = 0.038; R = 0.145). There were no significance differences in age among groups.

### 3.5. Correlations of Bite Force with Body Mass Index Values

**Figure 5:**
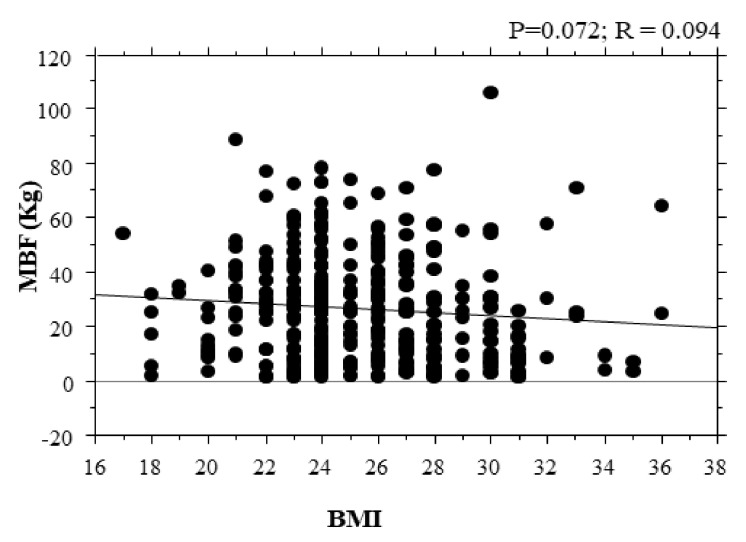
Correction of bite force with BMI. Correlations of maximal bite force with body mass index. MBF: maximal bite force; BMI: body mass index; R= coefficient of correlation; P = *p*-value (*p*).

Correlation of bite force with body mass index is shown in Figure 5. There was no correlation between bite force and BMI (*p* = 0.07; R = 0.094).

## 4. Discussion

The results obtained can be summarized as follows: (1) MBF is higher in males than females, regardless of the presence or absence of teeth. In patients with traditional CD or IRO prostheses, there are no differences between males and females; (2) the use of prostheses improves MBF compared to edentulous patients and this effect is greater with IRO prostheses; (3) the chewing force of patients with all natural teeth still remains greater; (4) there are no differences between patients’ chewing strength based on different weight categories obtained by BMI in any experimental group, although FD subjects have a reduced incidence of obesity; (5) there is a significant negative correlation between MBF and age, and no correlation between MBF and BMI, although, in the latter case, there is a trend towards a negative correlation (*p* = 0.07).

MBF is an important index to assess the functional status of the masticatory system. Every patient generates his own MBF, as highlighted by Eriksson [26], showing that the individual variability is due to the difference in fiber composition of masticatory muscle caused by masticatory habits (hard or soft food) and genetic influences. 

We observed a negative association between age and MBF. Many studies have reported the presence of a decrease in MBF with advancing age [27], an effect linked to many reasons, including the loss of masticatory muscle mass, the number of teeth or the condition of the dental arches. Nonetheless, we found that, in the elderly, the MBF is higher in males than in females. These data are independent of the patient’s condition (toothed or non-toothed) or the presence of different types of prostheses. It can, therefore, be deduced that males in old age start from an advantageous position compared to females as regards the chewing function. This finding is in line with other studies in elderly [28] or adult [29] patients and is likely due to increased chewing muscle mass in males.

The level of MBF is, however, influenced by the presence of one’s own teeth. In fact, patients of the FD group (with their own full dentition) have a much higher MBF than those with prostheses and patients without teeth. This finding is also confirmed by other studies that show that although the average MBF doubled after implant treatment in CD wearers patients, the highest MBF value was shown by dentate subjects [30,31,32].

Nonetheless, the use of dental prostheses improved chewing function compared to edentulous patients. These data underline the importance of the use of prostheses in elderly people to improve masticatory function and the quality of life. We found that the chewing force is greater in patients with IRO than in patients with traditional prostheses (CD/CD and CD/T groups). These data are actually confirmed by findings showing that people with full natural dentition or IRO wearers are more satisfied and have better quality of life than people wearing CD [7,30,33].

Our data show that, although the maintenance of the own tooth remains the optimal condition to maintain MBF in elderly, teeth replacement by means of implant-retained overdentures is a more reliable alternative than CD for edentulous patients. This finding is supported by many studies [7,30,34] showing that approximately half number of chewing cycles, to halve the initial size of a test food, is required for patients wearing IRO prostheses as compared to patients wearing CD. This results in beneficial effects on masseter muscle thickness, MBF, and masticatory efficiency, and prevents the progression of alveolar bone resorption [35,36]. 

The difference between the two types of prostheses can have several reasons. One reason may be the instability of the traditional dentures, or the psychological effect due to awareness of a more reliable structure. During mastication, occlusal forces in CD wearers are fully unloaded on the underlaying mucosa, often exceeding pain threshold of the mucosa and periosteum. In addition, the CD low elastic modulus and the denture displacement causes pain above a determined load, as happened during our test, and further ridge resorption. A reduced retention and the prosthesis dislodgment represent a limit to the chewing force [37]. These problems are emphasized during the MBF test because the mucoperiosteum pain prevent the closure force. In addition, the patient’s reluctance to cooperate may cause reduced values. Habitual lower muscular training results in muscle bulk loss and atrophy [38], as documented by computed tomography [14].

IRO prostheses made it possible to download occlusal forces that occurred directly on the bone, without causing any pain. Overdenture stabilization improves MBF and masticatory function in our patients, as reported by other authors [7,15,30,35,39] and promotes masseter thickness enlargement [35].

The MBF values observed in IRO group were higher than those reported in other studies [15,35]. The high values in our study might be attributable to the greater resiliency of telescopic attachment versus other methods like balls, bars and locators [22,23]. Telescopic attachments minimize movements of the distal portion of the denture, improve horizontal stability, and increase chewing efficiency and MBF, compared to other attachments [15]. In addition, it is possible that, in this study, the higher number of implants to support overdentures, four instead of two, may have influenced the MBF data. Multiple implants might indeed increase the support during chewing and minimize the occlusal load, especially in subjects with high bite force capability. Despite this, it has not been clearly demonstrated that a higher number of implants offers better outcomes or higher patient satisfaction than a smaller number of implants [35,40,41]. Furthermore, other authors did not observe significant improvement of chewing function after implant supported overdentures treatment [42].

It should be noted that the use of IRO prostheses does not bring the MBF values back to the levels of subjects with full dentition. One possible explanation is that the lower force in IRO wearers than in dentate subjects might be partially attributable to the loss of periodontal receptors, with consequent weakened reflexes and mandible movements, as suggested by other authors [33,34,43].

In our study, we did not find significant differences in MBF for the groups that had upper and lower CD (CD/CD) compared to the groups that had CD and their own teeth (CD/T). Although it is reasonable to assume that in the CD/T group occlusion is more stable, it is possible that the CD dislodgement and the pain the patients experience above a certain load could abolish the difference between subjects occluding between two CDs or between CD and their teeth. It is true that upper CD is more stable and guarantees higher MBF than lower CD. In this study, however, we did not distinguish between subjects wearing CD in the superior or inferior arch. This is a limitation of this study.

Maximum bite force (MBF) improves more in patients who use implant-retained overdentures than traditional complete dentures, but without reaching the values of subjects with full natural dentition. Although MBF changes do not seem to have an influence on body mass index, we found increased percentages of obesity in edentulous subjects or those with prostheses. Thus, it is advised that old people wearing traditional complete dentures need special attention by a nutritionist to avoid risk of malnutrition.

The individual chewing strength could affect the type of food consumed. Every food requires force to be penetrated that depends on his own consistency and hardness. Harder food requires higher muscular activity than soft food [7]. To ensure safe food mastication, bite force should be higher than the yield force required to fragment a food material [44]. In the elderly without teeth and with dentures, the chewing force is reduced compared to patients with dentition. These people have to chew for a longer time and swallow poorly chewed food. This fact could lead these patients towards a soft food diet rich in carbohydrates sugar and fats, with risk of malnutrition (due to the lack of meat, vegetables and fruits), despite weight gain. For this reason, we compared the BMI, an index of body fat based on height and weight, to the MBF of the various groups. We found no association between weight categories and chewing strength. In other words, there is no association between reduced chewing strength and weight gain due to different food consumption. Nonetheless, we observed that subjects with full dentition, and therefore with higher MBF, have a lower incidence of obesity (5%) than subjects of the other experimental groups whose average percentage of obesity is around 18–22%.

We cannot exclude that the inclusion of a large number of subjects with natural teeth compared to the other groups may have hidden a correlation between weight and MBF. However, it must be said that MBF is a voluntary effort requested by the patient that last only for 5 s, whereas lower forces are used during normal mastication. Therefore, MBF value does not reflect the real habitual masticatory force used to grind and swallow food. Gibbs and coworkers [45] claim that masticatory forces during chewing and swallowing of dentate subjects are about 40% of their MBF. Therefore, it is possible that even patients with reduced MBF can take advantage of a fairly varied and balanced diet.

In Table 1, we have tried to depict the masticatory force of the subjects in relation to the force required to disrupt the food. In some studies, mastication in relation to hard or soft food has been investigated [7,46,47].

In the study of Eerikäinen and Könönen [46], it was reported that the greatest force is required to penetrate rye bread (167 N–17 kg) followed by raw carrot (118 N–12 kg), boiled meat (80 N–8 kg), raw cabbage (74 N–7.5 kg), and cooked meat (124 N–12.5 kg). Thus, assuming that 40% of MBF represents the real masticatory force [45], we have distinguished between people with 40% masticatory force greater and lower than 8 kg, an arbitrary value that can provide an indication of the type of food that can be masticated.

As shown in the table, 92% of subjects in the FD group develop a chewing force greater than 8 kg (required, for example, to chew and swallow boiled meat) and are able to crunch foods that are necessary to guarantee a satisfactory nutrition. This is in line with the finding that FD subjects have a significantly lower percentage of obesity as compared to the other groups. Therefore, it seems that people with sufficient chewing strength can, in theory, feed more adequately. In the IRO group, 62.5% of subjects (65% males and 60% females) are able to exceed 8 kg during normal mastication and may aspire to a complete and satisfying diet. In the CD/T group, only 4.5% of males exceed 8 kg, whereas none of groups CD/CD and ED are able to penetrate foods like boiled meat, vegetables and carrots. As a consequence, people of the last two groups have to chew longer and have to swallow poorly chewed food. These subjects may prefer soft food that is rich in carbohydrates, sugar and fats, with risk of weight gain and malnutrition due to the lack of meat, vegetables and fruits.

Although this calculation is arbitrary and it has only an indicative value, it gives an idea of the real difficulty that elderly people wearing prostheses have during mastication. The improvement of maximum bite force and chewing efficiency may be an important prerequisite for adequate nutrition. The problem of malnutrition has been highlighted recently in some articles that explain how the elderly become malnourished due to problems also related to prosthesis [5,33]. It is clear that these patients require special attention from a nutritionist in order to be able to eat properly. This aspect is probably often overlooked by geriatricians who also need to pay attention to balance the diet, to avoid fattening and to identify whether people need nutritional supplements of proteins.

## 5. Conclusions

In conclusion, this study showed that MBF improves more in patients who use IRO prostheses than traditional ones, but without reaching the values of subjects with full natural dentition. Nonetheless, the use of IRO prostheses is recommended to achieve a significant improvement in MBF in elderly patients when compared to traditional complete dentures. Although MBF changes do not seem to have an influence on BMI, we found increased percentages of obesity in edentulous subjects or those with prostheses. Thus, it is advised that old people wearing traditional complete dentures need special attention by a nutritionist to avoid risk of malnutrition.

## Figures and Tables

**Table 1:** Comparison of the means of maximum bite force (MBF) among the experimental groups. Assuming that 40% of MBF represents the real masticatory force, we have distinguished between people with 40% masticatory force greater and lower than 8 kg, an arbitrary value that can provide an indication of the type of food that can be masticated.

Experimental Groups	Gender	MBF Max (Right/Left)	MBF Min (Right/Left)	Mean MBF	Total Mean MFB	No. Subject 40% < 8 kg	%	No. Subject 40% > 8 kg	%	Mean %
Full dentition (FD)	males	106	15.5	44.8	39.7	7	6.6	98	94.5	92.2
	females	65.5	11.5	34.6		10	10	90	90	
Overdentures/overdentures (IRO)	males	31	8.5	22.4	21.7	7	35	13	65	62.5
	females	28	12.5	21.1		8	40	12	60	
Conventional prosthesis/teeth (CD/T)	males	20.5	6.5	11.6	10.8	21	95.4	1	4.5	2.25
	females	14	6	10		0				
Conventional prosthesis in both arches (CD/CD)	males	11.5	4	7.7	7.4	0				
	females	11.5	3.5	7.2		0				
Edentulous patients (ED)	males	4.5	1.5	2.7	2.3	0				
	females	3	1	2		0				

## Data Availability

The data presented in this study are available on request from the corresponding author.
